# Supplementary material for: Factors affecting the number of influenza patients before and during COVID-19 pandemic, Thailand
Source: PLoS One. 2024 May 10;19(5):e0303382. doi: 10.1371/journal.pone.0303382 (PMC11086856; doi:10.1371/journal.pone.0303382)
Supplement: S1 Table — (PDF) [file pone.0303382.s001.pdf]

S1_Table: Data retrieved and its source.

Sources	Data (variables)	The dates when the data was accessed in this study
Thai meteorological department	(1) Daily rainfall by province<br>(2) Number of rainy days by province<br>(3) Relative humidity by day by province<br>(4) Daily temperature by province<br>(5) Seasonality by province	12 October 2021
Thailand national statistics office	(6) Annual income per household by province	30 December 2021
Epidemiology Division, Department of Disease Control	(7) Daily reported influenza patients through national disease notification surveillance system	23 November 2021
Strategy and planning division, Ministry of Public Health	(8) Mid-year population by province	17 October 2021
Energy policy and planning office, Ministry of Energy	(9) Total area (square kilometers) by province	26 July 2021
Center for COVID-19 Situation Administration, Ministry of Interior (https://www.moicovid.com/)*	(10) Public health control measures by province (monthly regulated: yes/ no)<br>10.1 Prohibition of travelling to restricted area (Lock down)<br>10.2 Border closure to Kingdom of Thailand	2 July 2021

Sources	Data (variables)	The dates when the data was accessed in this study
	10.3 Travel restriction at specific time (Curfew)<br>10.4 Prohibition of group gatherings<br>10.5 Restriction of travelling across provinces<br>10.6 School measures and closures	

Abbreviations: *, available at the time of COVID-19 pandemic, but not available at present
