# Supplementary material for: Factors affecting the number of influenza patients before and during COVID-19 pandemic, Thailand
Source: PLoS One. 2024 May 10;19(5):e0303382. doi: 10.1371/journal.pone.0303382 (PMC11086856; doi:10.1371/journal.pone.0303382)
Supplement: S2 Table — (PDF) [file pone.0303382.s002.pdf]

S2_Table: Factors associated with number of influenza patients during 2014 – 2019

Factors	Univariable analysis		Multivariable analysis	
	β (95% CI)[&]	*p*-value	adj. β (95% CI)[&]	*p*-value
Average monthly rainfall	0.19 (0.10, 0.28)	<0.001	-	-
Number of rainy days per month	3.93 (2.11, 5.75)	<0.001	-	-
Average relative humidity per month	5.64 (3.64, 7.63)	<0.001	-	-
Average monthly temperature	-3.37 (-10.68, 3.93)	0.366	-	-
Average monthly income per household	-0.01 (-0.02, > -0.01)	<0.001	-0.20 (-0.03, -0.01)	<0.001
Population density per square kilometer	0.68 (0.54, 0.82)	<0.001	1.00 (0.82, 1.18)	<0.001
Seasonality (monthly)		<0.001		<0.001
summer [ref.]				
rainy	119.96 (90.35, 149.57)		137.15 (86.17, 188.13)	
winter	46.72 (16.21, 77.22)		56.46 (3.21, 109.71)	

Abbreviations: β, Regression coefficient; adj. β, adjusted regression coefficient; 95% CI, 95% confidence interval; ref., reference group; *p*-value, *p*-value from the Wald test.
