# Supplementary material for: Factors affecting the number of influenza patients before and during COVID-19 pandemic, Thailand
Source: PLoS One. 2024 May 10;19(5):e0303382. doi: 10.1371/journal.pone.0303382 (PMC11086856; doi:10.1371/journal.pone.0303382)
Supplement: S3 Table — (PDF) [file pone.0303382.s003.pdf]

S3_Table: Factors associated with number of influenza patients during 2020 – 2021

Factors	Univariable analysis		Multivariable analysis	
	$\beta^{\#}$ (95% CI)$^{\&}$	*p*-value	$\beta^{\#}$ (95% CI)$^{\&}$	*p*-value
Average monthly rainfall	-0.27 (-0.45, -0.10)	0.002	-	-
Number of rainy days per month	-8.03 (-11.55, -4.52)	<0.001	-	-
Average relative humidity per month	-8.96 (-12.02, -5.89)	<0.001	-	-
Average monthly temperature	-14.83 (-25.93, -3.72)	0.009	-	-
Population density per square kilometer	0.20 (0.15, 0.26)	<0.001	0.20 (0.15, 0.26)	<0.001
Seasonality (monthly)		<0.001		<0.001
summer [ref.]				
rainy	-14.38 (-69.05, 40.27)		-164.23 (-229.93, -98.52)	
winter	169.93 (115.34, 224.52)		61.06 (0.71, 121.41)	
Prohibition of travelling to restricted areas (lock down)		<0.001		<0.001
Not available [ref.]				
Available	-156.77 (-202.37, -111.16)		-169.34 (-233.52, -105.16)	
Restriction of travelling across provinces		<0.001		0.024
Not available [ref.]				
Available	-128.42 (-175.02, -81.82)		-66.88 (-125.15, -8.62)	
Border closure to Kingdom of Thailand.		0.071	-	-

Factors	Univariable analysis		Multivariable analysis	
	$\beta^{\#}$ (95% CI)$^{\&}$	*p*-value	$\beta^{\#}$ (95% CI)$^{\&}$	*p*-value
Not available [ref.]				
Available	-49.96 (-104.17, 4.24)			
Travel restriction at specific time (Curfew)		0.003	-	-
Not available [ref.]				
Available	-92.01 (-152.37, -31.65)			
Prohibition of group gatherings		<0.001	-	-
Not available [ref.]				
Available	-183.02 (-229.83, -136.21)			
School measures and closures		<0.001	-	-
Not available [ref.]				
Available	-140.47 (-185.83, -95.11)			

Abbreviations: β, Regression coefficient; adj. β, adjusted regression coefficient; 95% CI, 95% confidence interval; ref., reference group; *p*-value, *p*-value from the Wald test.
