# Supplementary material for: Factors affecting the number of influenza patients before and during COVID-19 pandemic, Thailand
Source: PLoS One. 2024 May 10;19(5):e0303382. doi: 10.1371/journal.pone.0303382 (PMC11086856; doi:10.1371/journal.pone.0303382)
Supplement: Attachment — Submitted filename: Response to Reviewers.pdf [file pone.0303382.s005.pdf]

Response to Reviewers [PONE-D-23-31302]

Dear Reviewers,

Reviewer#1: Thank you so much for your kind support to our study as shown in your precious comments to the Author.

Reviewer#2: Thank you so much for your precious comments indicating that the manuscript needs further clarification, and all responses are in the table below.

No.	Points from Reviewer#2	Author Responses
1.	The study doesn't seem replicable	The study can be replicated when the variables are available which can be further analyzed.
2.	There is need to define the range for the level of income in the data/result<br>Suggest, a population of each province be made available to get a better picture of the cases	2.1 The range of incomes are included in line 177 - 184.<br>2.2 Population density data is available as attached with the first submission (Data_2014-2021_24Sep23.xls).<br>2.3 Thank you for the remark on this variable (household income) which we can add in the study limitation in line 288-291.
3.	The influenzas cases were confirmed using what diagnostic method? needs to be stated	It is described in line 105-106.

Thank you so much for your further revision.

Best regards,

Pornthip Chompook

6 April 2024
